# Timing the SARS-CoV-2 index case in Hubei province

**DOI:** 10.1126/science.abf8003

**Published:** 2021-03-18

**Authors:** Jonathan Pekar, Michael Worobey, Niema Moshiri, Konrad Scheffler, Joel O. Wertheim

**Affiliations:** 1 Bioinformatics and Systems Biology Graduate Program, University of California San Diego, La Jolla, CA 92093, USA.; 2 Department of Biomedical Informatics, University of California San Diego, La Jolla, CA 92093, USA.; 3 Department of Ecology and Evolutionary Biology, University of Arizona, Tucson, AZ 85721, USA.; 4 Department of Computer Science and Engineering, University of California San Diego, La Jolla, CA 92093, USA.; 5 Illumina, Inc., San Diego, CA 92122, USA.; 6 Department of Medicine, University of California San Diego, La Jolla, CA 92093, USA.

## Abstract

Understanding when severe acute respiratory syndrome coronavirus 2 (SARS-CoV-2) emerged is critical to evaluating our current approach to monitoring novel zoonotic pathogens and understanding the failure of early containment and mitigation efforts for COVID-19. We used a coalescent framework to combine retrospective molecular clock inference with forward epidemiological simulations to determine how long SARS-CoV-2 could have circulated before the time of the most recent common ancestor of all sequenced SARS-CoV-2 genomes. Our results define the period between mid-October and mid-November 2019 as the plausible interval when the first case of SARS-CoV-2 emerged in Hubei province, China. By characterizing the likely dynamics of the virus before it was discovered, we show that more than two-thirds of SARS-CoV-2like zoonotic events would be self-limited, dying out without igniting a pandemic. Our findings highlight the shortcomings of zoonosis surveillance approaches for detecting highly contagious pathogens with moderate mortality rates.

## Backtracking a pandemic

Severe acute respiratory syndrome coronavirus 2 (SARS-CoV-2) may have had a history of abortive human infections before a variant established a productive enough infection to create a transmission chain with pandemic potential. Therefore, the Wuhan cluster of infections identified in late December of 2019 may not have represented the initiating event. Pekar *et al.* used genome data collected from the early cases of the COVID-19 pandemic combined with molecular clock inference and epidemiological simulation to estimate when the most successful variant gained a foothold in humans. This analysis pushes human-to-human transmission back to mid-October to mid-November of 2019 in Hubei Province, China, with a likely short interval before epidemic transmission was initiated.

*Science*, this issue p. 412

## Teaser

SARS-CoV-2 human-to-human transmission likely initiated in mid-October to mid-November 2019 in Hubei, China.

In late December of 2019, the first cases of COVID-19, the disease caused by severe acute respiratory syndrome coronavirus 2 (SARS-CoV-2), were described in the city of Wuhan in Hubei province, China (*1*, *2*). The virus quickly spread within China (*3*). The cordon sanitaire that was put in place in Wuhan on 23 January 2020 and mitigation efforts across China eventually brought about an end to sustained local transmission. In March and April 2020, restrictions across China were relaxed (*4*). By then, however, COVID-19 was a pandemic (*5*).

A concerted effort has been made to retrospectively diagnose the earliest cases of COVID-19 and thus determine when the virus first began transmitting among humans. Both epidemiological and phylogenetic approaches suggest an emergence of the pandemic in Hubei province at some point in late 2019 (*2*, *6*, *7*). The first described cluster of COVID-19 was associated with the Huanan Seafood Wholesale Market in late December 2019, and the earliest sequenced SARS-CoV-2 genomes came from this cluster (*8*, *9*). However, this market cluster is unlikely to have denoted the beginning of the pandemic, as COVID-19 cases from early December lacked connections to the market (*7*). The earliest such case in the scientific literature is from an individual retrospectively diagnosed on 1 December 2019 (*6*). Notably, however, newspaper reports document retrospective COVID-19 diagnoses recorded by the Chinese government going back to 17 November 2019 in Hubei province (*10*). These reports detail daily retrospective COVID-19 diagnoses through the end of November, suggesting that SARS-CoV-2 was actively circulating for at least a month before it was discovered.

Molecular clock phylogenetic analyses have inferred the time of the most recent common ancestor (tMRCA) of all sequenced SARS-CoV-2 genomes to be in late November or early December 2019, with uncertainty estimates typically dating to October 2019 (*7*, *11*, *12*). Crucially, though, this tMRCA is not necessarily equivalent to the date of zoonosis or index case infection (*13*, *14*) because coalescent processes can prune basal viral lineages before they have the opportunity to be sampled, potentially pushing SARS-CoV-2 tMRCA estimates forward in time from the index case by days, weeks, or months. For a point of comparison, consider the zoonotic origins of the HIV-1 pandemic, whose tMRCA in the early 20th century coincided with the urbanization of Kinshasa, in what is now the Democratic Republic of the Congo (*15*, *16*), but whose cross-species transmission from a chimpanzee reservoir occurred in southeast Cameroon, likely predating the tMRCA of sampled HIV-1 genomes by many years (*17*). Despite this important distinction, the tMRCA has been frequently conflated with the date of the index case infection in the SARS-CoV-2 literature (*7*, *18*, *19*).

Here, we combine retrospective molecular clock analysis in a coalescent framework with a forward compartmental epidemiological model to estimate the timing of the SARS-CoV-2 index case in Hubei province. The inferred dynamics during these unobserved early days of SARS-CoV-2 highlight challenges in detecting and preventing nascent pandemics.

**Fig. 1:**
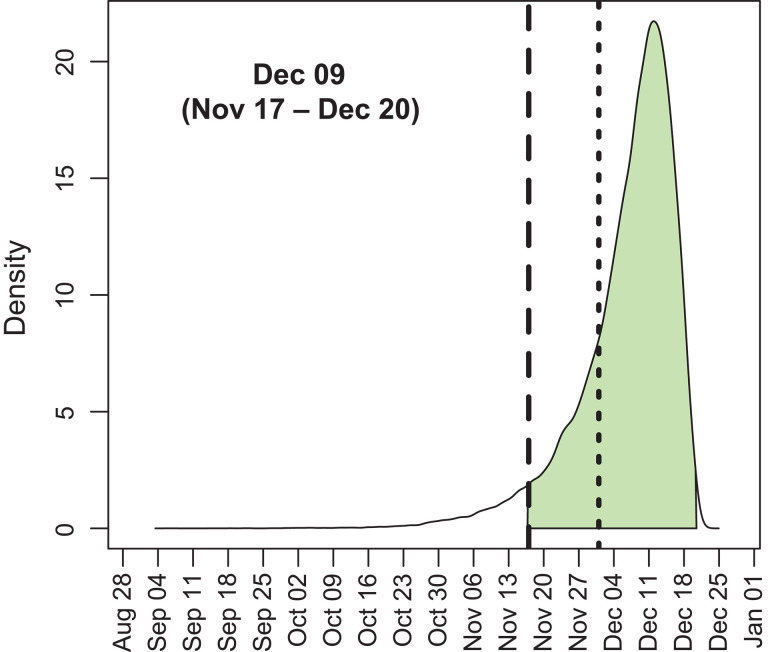
Posterior distribution for the tMRCA of 583 sampled SARS-CoV-2 genomes circulating in China between December 2019 and April 2020. Inference was performed by using a strict molecular clock and a Bayesian Skyline coalescent prior. The shaded area denotes 95% HPD. The long-dashed line represents 17 November 2019, and the short-dashed line represents 1 December 2019.

We first explored the evolutionary dynamics of the first wave of SARS-CoV-2 infections in China. We used Bayesian phylodynamics (*20*) to reconstruct the underlying coalescent processes using a Bayesian Skyline approach for 583 SARS-CoV-2 complete genomes, sampled in China between when the virus was first discovered at the end of December 2019 and the last of the non-reintroduced circulating virus in April 2020. Applying a strict molecular clock, we inferred an evolutionary rate of 7.90 10^4^ substitutions per site per year [95% highest posterior density (HPD): 6.64 10^4^ to 9.27 10^4^]. The tMRCA of these circulating strains was inferred to fall within a 34-day window with a mean of 9 December 2019 (95% HPD: 17 November to 20 December) (Fig. 1). This estimate accounts for the many disparate inferred rooting orientations [see supplementary text and (*21*)]. Notably, 78.7% of the posterior density postdates the earliest published case on 1 December, and 95.1% postdates the earliest reported case on 17 November. Relaxing the molecular clock provides a similar tMRCA estimate, as does applying a Skygrid coalescent approach (fig. S1). The recency of this tMRCA estimate in relation to the earliest documented COVID-19 cases obliges us to consider the possibility that this tMRCA does not capture the index case and that SARS-CoV-2 was circulating in Hubei province before the inferred tMRCA.

**Fig. 2:**
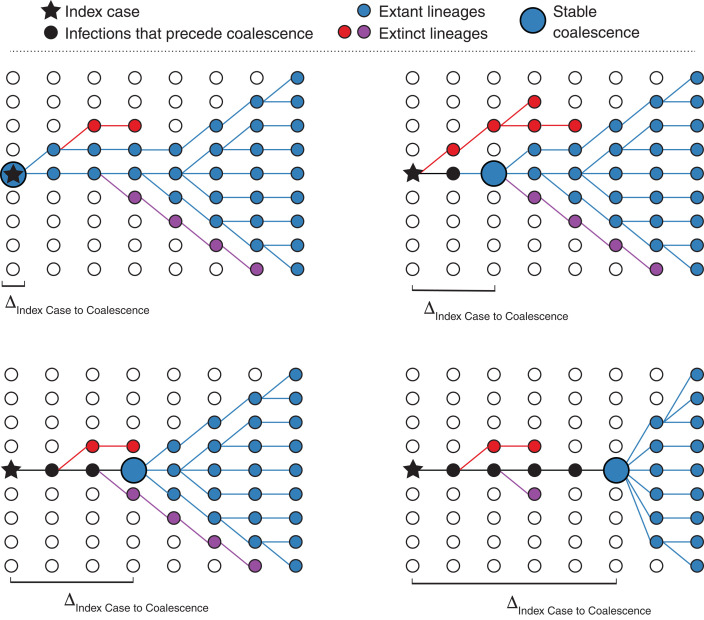
Hypothetical coalescent scenarios depicting how the time between index case infection and time of stable coalescence can vary on the basis of stochastic extinction events of basal viral lineages. Coalescence can occur within or contemporaneously with the index case (upper left) or, in cases infected later in the course of the epidemic, with one (upper right) or more (lower left) basal lineages going extinct. In extreme cases, the epidemic can persist at low levels for a long time before stable coalescence (lower right).

If the tMRCA postdates the earliest documented cases, then the earliest diverged SARS-CoV-2 lineages must have gone extinct (Fig. 2). As these early basal lineages disappeared, the tMRCA of the remaining lineages would move forward in time (fig. S2). Thus, we interrogated the posterior trees sampled from the phylodynamic analysis to determine whether this time of coalescence had stabilized before the sequencing of the first SARS-CoV-2 genomes on 24 December 2019 or whether this process of basal lineage loss was ongoing in late December and/or early January. Notably, these basal lineages need not be associated with specific mutations, as the phylodynamic inference reconstructs the coalescent history, not the mutational history (*20*).

We find only weak evidence for basal lineage loss between 24 December 2019 and 13 January 2020 (fig. S3A). The root tMRCA is within 1 day of the tMRCA of virus sampled on or after 1 January 2020 in 78.5% of posterior samples (fig. S3B). The tMRCA of genomes sampled on or after 1 January 2020 is 3 days later than the tMRCA of all sampled genomes. By contrast, the mean tMRCA does not change when considering genomes sampled on or after 1 January 2020 versus on or after 13 January 2020. This consistency indicates a stabilization of coalescent processes at the start of 2020, when an estimated total of 1000 people had been infected with SARS-CoV-2 in Wuhan (*22*). Nonetheless, to account for the weak signal of a delay in reaching a stable coalescence (i.e., the point in time at which basal lineages cease to be lost), we identified the tMRCA for all viruses sampled on or after 1 January 2020 (i.e., at the time of stable coalescence) for each tree in the posterior sample.

Phylogenetic analysis alone cannot tell us how long SARS-CoV-2 could have circulated in Hubei province before the tMRCA. To answer this question, we performed forward epidemic simulations (*23*). These simulations were initiated by a single index case using a compartmental epidemiological model across scale-free contact networks (mean number of contacts: 16). This compartmental model was previously developed to describe SARS-CoV-2 transmission dynamics in Wuhan (*22*). This model, termed SAPHIRE, includes compartments for susceptible (S), exposed (E), presymptomatic (P), unascertained (A), ascertained (I), hospitalized (H), and removed (R) individuals. Our simulations used parameters from the time period before COVID-19 mitigation efforts, from 1 January through 22 January 2020 (table S1), based on the work of Hao *et al*. (*22*). We analyzed 1000 epidemic simulations that resulted in 1000 total infected people. These simulated epidemics had a median doubling time of 4.1 days (95% range across simulations: 2.7 to 6.7), matching premitigation incidence trends in Wuhan (table S2).

**Fig. 3:**
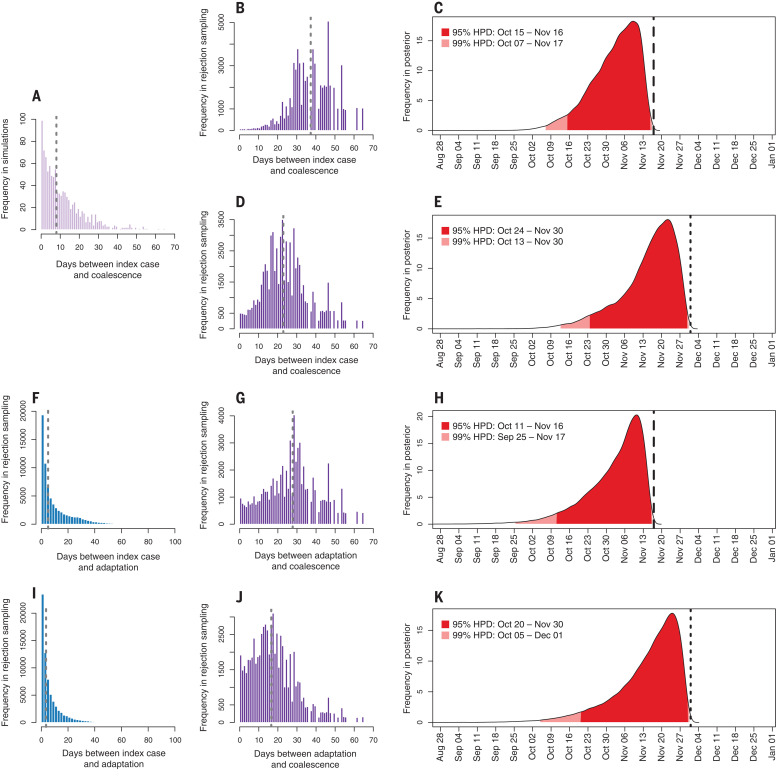
Forward simulations estimating the timing of the index case in Hubei province. (**A**) Days between index case infection and stable coalescence in forward compartmental epidemic simulations (*n* = 1000). (**B** and **C**) Days between index case infection and stable coalescence after rejection sampling (B) and posterior distribution for date of index case infection (C), conditioned on an ascertained case by 17 November 2019, which is denoted by a long-dashed line. (**D** and **E**) Epidemic simulation, conditioned on an ascertained case by 1 December 2019, which is denoted by a short-dashed line. (**F** to **H**) Two-phase epidemic (F) days between index case carrying less-fit variant and adaptation (*n* = 2000), (G) days between adaptation and stable coalescence (*n* = 1000), and (H) posterior distribution for date of index case infection, conditioned on an ascertained case by 17 November 2019. (**I** to **K**) Two-phase epidemic conditioned on an ascertained case by 1 December 2019. Gray dashed lines indicate median estimates.

We simulated coalescent processes across the transmission network to determine the tMRCA of the virus at the end of the simulation. This approach allowed us to determine the distribution of the expected number of days between index case infection and the stable coalescence (i.e., tMRCA) (Fig. 2). The median number of days between index case infection and this tMRCA was 8.0 days (95% range: 0.0 to 41.5 days) (Fig. 3A). The median time between index case infection and the first person exiting the presymptomatic phase (i.e., ascertained or unascertained infection) was 5.7 days (95% range: 0.9 to 15.7 days).

As a robustness check, we also simulated epidemics with more densely and more sparsely connected contact networks (mean: 26 and 10 contacts, respectively), rescaling the per-contact transmission rate to maintain empirical epidemic growth dynamics. We also explored the effects of faster (mean: 3.1 days; 95% range: 2.0 to 5.1 days) and slower (mean: 5.3 days; 95% range: 3.6 to 7.5 days) epidemic doubling times (table S2). Slower transmission rates led to more days between the index case and the stable coalescence, but modifying the density of the contact network had minimal effect on this time interval (fig. S4).

To estimate the date of infection for the index case in Hubei province, we combined the retrospective molecular clock analysis with the forward epidemic simulations (fig. S5). We identified the stable tMRCA in the posterior trees as an anchor to the real-world calendar dates and then extended this date back in time according to the number of days between the index case infection and the time of stable coalescence from the compartmental epidemic simulations. However, a random sample of tMRCAs and days from index case infection to coalescence will not produce epidemiologically meaningful results because many of these combinations do not precede the earliest dates of reported COVID-19 cases. Therefore, we implemented a rejection samplingbased approach to generate a posterior distribution of dates of infection for the Hubei index case, conditioning on at least one individual who had progressed past the presymptomatic stage in the simulated epidemic before the date of the first reported COVID-19 case (see materials and methods and fig. S6).

In our primary analysis, we assume that 17 November represents the first documented case of COVID-19 (ascertained or unascertained in the SAPHIRE model). Under this assumption, the median number of days between index case infection and stable coalescence after rejection sampling is 37 days (95% HPD: 12 to 55 days) (Fig. 3B). Consequently, the index case in Hubei likely contracted SARS-CoV-2 on or around 4 November 2019 (95% upper HPD: 15 October; 99% upper HPD: 7 October) (Fig. 3C).

This time frame for the Hubei index case is robust (fig. S7). Epidemic simulations with faster or slower transmission rates and more or less densely connected contact networks produce similar date estimates. Furthermore, using the root tMRCA from the sampled posterior trees, rather than adjusting for the shifting coalescence between 24 December 2019 and 1 January 2020, produces a median date of 3 November (95% upper HPD: 14 October; 99% upper HPD: 4 October). Incorporating a relaxed molecular clock or adjusting the coalescent prior assumption (Skyline versus Skygrid) also had minimal effect.

If we enforce that there must be at least one ascertained case in our simulations before 17 November 2019, the median date of the Hubei index case is pushed back about a week to 28 October (95% upper HPD: 11 October; 99% upper HPD: 5 October) (fig. S7). However, the distinction between ascertained and unascertained in the original SAPHIRE model was meant to reflect the probability of missed diagnoses in January 2020 of the Wuhan epidemic and does not account for the investigations that resulted in retrospective diagnoses in November and December 2019.

If we discount the reported evidence of retrospective COVID-19 diagnoses throughout the end of November and instead take 1 December as representing the first confirmed case of COVID-19, then the median time between index case infection and stable coalescence after rejection sampling is 23 days (95% range: 1 to 47 days) (Fig. 3D). Under this scenario, the index case in Hubei would have contracted SARS-CoV-2 on or around 17 November 2019 (95% upper HPD: 24 October; 99% upper HPD: 13 October) (Fig. 3E). Similar dates are inferred with a relaxed molecular clock and conditioning on an ascertained infection by 1 December (fig. S7).

It is reasonable to postulate that the variant of SARS-CoV-2 that first emerged was less fit than the variant that spread through China and that evolutionary adaptation was critical to its establishment in humans (*12*). Therefore, we simulated two-phase epidemics in which the index case was infected with a less-fit variant (i.e., half as transmissible) that went extinct, but not before giving rise to a mutant strain matching the transmission dynamics estimated in Wuhan (figs. S8 and S9). If we condition on an ascertained or unascertained COVID-19 case (due to either the original or adapted variant) by 17 November, the original variant transmits for a median of 5 days (95% HPD: 0 to 36 days) before the adapted strain emerges (Fig. 3F); this adapted strain then circulates for a median of 28 days (95% HPD: 0 to 52 days) before reaching a stable coalescence (Fig. 3G). In this scenario, the index case in Hubei would have likely contracted an unobserved variant of SARS-CoV-2 on 5 November 2019 (95% upper HPD: 11 October; 99% upper HPD: 25 September) (Fig. 3H). If we again discount the reported evidence of COVID-19 in November and take 1 December as the first confirmed case, then the virus would spend less time in humans across both phases of the early epidemic (Fig. 3, I and J), and the index case in Hubei would have acquired SARS-CoV-2 on 18 November 2019 (95% upper HPD: 20 October; 99% upper HPD: 5 October) (Fig. 3K). As in the primary analysis, the inferred date of the index case in the two-phase epidemic was robust to varying model assumptions (fig. S10), including the amount by which viral fitness differed between the two phases (supplementary text and fig. S18).

These one-phase and two-phase epidemic simulations both suggest that the tMRCA is not representative of the emergence of SARS-CoV-2. In the primary analysis, the index case remains infected at the tMRCA (as in Fig. 2, upper left panel) in <1.1% of simulated epidemics (table S3). In the two-phase epidemics, the index case is still infected at the tMRCA in 2.2% of simulations, likely because of an increased variance in the time between index case and tMRCA. The initial, less-fit variant persisted until the tMRCA in 17% of simulated epidemics and until 1 January 2020 in 3.7% of simulated epidemics. However, when this less-fit variant persisted, it was represented by only a single infected individual at the tMRCA; this low frequency suggests that even if this less-fit variant did exist, it could have easily been missed in early genome sequencing efforts. In the two-phase epidemic, the first ascertained (or unascertained) case was due to the less-fit variant in around two-thirds of simulated epidemics.

**Fig. 4:**
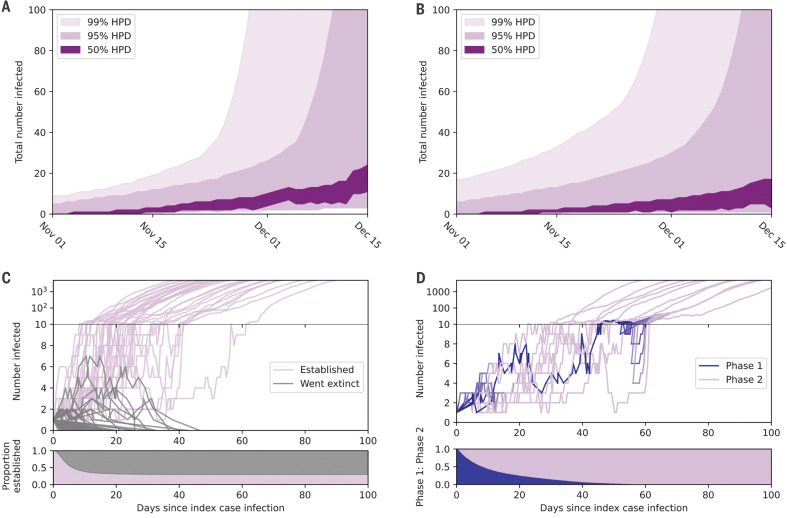
Epidemic growth in compartmental simulations. (**A**) Estimated total number of people infected in late 2019. Dark purple shading represents central 50% HPD, intermediate purple shading represents central 95% HPD, and light purple represents central 99% HPD. (**B**) Estimated total number of people infected in late 2019 for a two-phase epidemic. (**C**) Number of people infected over time in a sample of epidemic simulations that established (purple; *n* = 30) and went extinct (gray; *n* = 70). The *y* axis transitions to log scale once 10 people are infected at any given time. The lower panel shows the proportion of simulations that still have at least 1 infected individual over time (persisting epidemics in purple; extinct epidemics in gray). (**D**) Sample (*n* = 10) of two-phase epidemic simulations transitioning from less-fit phase 1 (blue) to more-fit phase 2 (purple). Each line represents a single simulation and its transition over time. The lower panel shows the average proportion of phase 1 to phase 2infected individuals over time.

By anchoring our epidemic simulations to specific tMRCA estimates, we can reconstruct a plausible range for the number of SARS-CoV-2 infections before the discovery of the virus (Fig. 4A). The median number of individuals infected with SARS-CoV-2 in our primary analysis is less than one until 4 November. The median number of infected individuals is four (95% HPD: 1 to 13) on 17 November and reaches nine (95% HPD: 2 to 26) on 1 December. These values are generally robust to model specifications, molecular clock method, and date of first COVID-19 case (table S4 and fig. S11). The two-phase epidemics tend to exhibit similar growth patterns (Fig. 4B, fig. S12, and table S5). Notably, we do not see any evidence for an increase in hospitalizations until mid- to late December, even when we increase the virulence of the less-fit variant in the two-phase epidemics or increase the probability of hospitalization before the stable coalescence (supplementary text and figs. S13 and S14).

Empirical observation throughout the SARS-CoV-2 pandemic has shown the outsized role of superspreading events in the propagation of SARS-CoV-2 (*24*,*27*), wherein the average infected person does not transmit the virus. Our results suggest that the same dynamics likely influenced the initial establishment of SARS-CoV-2 in humans, as only 29.7% of simulated epidemics from the primary analysis went on to establish self-sustaining epidemics. The remaining 70.3% of epidemics went extinct (Fig. 4C). Simulated epidemics that went extinct typically produced only 1 infection (95% range: 1 to 9) and never more than 44 infections total or 14 infections at any given time (table S2). The median failed epidemic went extinct by day 8. As the contact network became more or less densely connected, the number of epidemics that went extinct was similar: 68.3 and 69.4%, respectively (table S2). However, the percentage of extinct epidemics increased as the transmission rate decreased (80.5%) and decreased as the transmission rate increased (53.6%). In the two-phase epidemic, this original less-fit variant went extinct by day 9 (95% HPD: 2 to 52) and produced a median of one infection (95% HPD: 1 to 13) (Fig. 4D and table S6).

The overdispersed nature of SARS-CoV-2 transmission patterns favors its persistence, as epidemics simulated over random contact networks (with the same mean number of contacts) that are not characterized by superspreading events (*27*) tended to go extinct more frequently, 83.7% of the time. Furthermore, the large and highly connected contact networks characterizing urban areas seem critical to the establishment of SARS-CoV-2. When we simulated epidemics in which the number of connections was reduced by 50 or 75% (without rescaling per-contact transmissibility) to reflect emergence in a rural community, the epidemics went extinct 94.5 or 99.6% of the time, respectively.

Our results highlight the unpredictable dynamics that characterized the earliest days of the COVID-19 pandemic. The successful establishment of SARS-CoV-2 postzoonosis was far from certain, as more than two-thirds of simulated epidemics quickly went extinct. It is highly probable that SARS-CoV-2 was circulating in Hubei province at low levels in November 2019 and possibly as early as October 2019, but not earlier. Nonetheless, the inferred prevalence of this virus was too low to permit its discovery and characterization for weeks or months. By the time that COVID-19 was first identified, the virus had firmly established itself in Wuhan. This delay highlights the difficulty in surveillance for novel zoonotic pathogens with high transmissibility and moderate mortality rates.

The high extinction rates we inferred suggest that spillover of SARS-CoV-2like viruses may be frequent, even if pandemics are rare (*28*). Furthermore, the same dynamics that characterized the establishment of SARS-CoV-2 in Hubei province may have played out all over the world, as the virus was repeatedly introduced but only occasionally took hold (*29*, *30*). The reports of cases in December 2019 and January 2020 in France and California that did not establish sustained transmission fit this pattern (*31*,*33*). However, our results suggest that polymerase chain reaction evidence of SARS-CoV-2 in wastewater outside of China before November 2019 is unlikely to be valid (*34*), and the suggestion of international spread in mid-November or early December 2019 should be viewed with skepticism (*35*,*37*), given that our results suggest that fewer than 20 people were infected with SARS-CoV-2 at this time (table S4 and fig. S11). Our results also refute claims (*38*) of large numbers of patients requiring hospitalization because of COVID-19 in Hubei province before December 2019 (figs. S13 and S14). Nevertheless, SARS-CoV-2 may be detectable in archived wastewater samples or other biomaterials from Hubei province from early to mid-November 2019, should they exist, and incorporating these types of data in our model could further refine our timing estimates. Moreover, wastewater detection may present the best chance of early detection of future pandemics during the early phase of spread for which we estimate very low numbers of infections (*39*).

Even though all of the earliest documented cases of COVID-19 were found in Hubei province, we cannot discount the possibility that the index case initially acquired the virus elsewhere. Nonetheless, our dating inference is insensitive to geography. Furthermore, our results suggest that if the virus first emerged in a rural community, it would have needed to migrate to an urban setting to avoid extinction. The lack of reports of COVID-19 elsewhere in China in November and early December suggests that Hubei province is the location where human-to-human transmission chains were first established.

The circumstances surrounding the emergence of SARS-CoV-2 in Hubei province remain shrouded. Although SARS-CoV-2 is repeatedly adapting to spread among humans (*40*, *41*), our findings do not reveal whether the virus that first emerged was less fit than the virus that spread throughout China. Nevertheless, the inferred timing of the index case is generally similar in both of these scenarios because less-fit viruses in our simulations that went extinct tended to do so very quickly. It is yet unknown whether the virus emerged directly from its animal reservoir [presumably horseshoe bats (*42*, *43*)] or first circulated in and possibly adapted to an intermediate host. Our estimates for the timing of the Hubei index case further distance this individual from the outbreak at the Huanan Seafood Wholesale Market. Finding the animal reservoir or hypothetical intermediate host will help to further narrow down the date, location, and circumstances of the original SARS-CoV-2 infection in humans. However, even in the absence of that information, coalescent-based approaches permit us to look back beyond the tMRCA and toward the earliest days of the COVID-19 pandemic. Although there was a pre-tMRCA fuse to the COVID-19 pandemic, it was almost certainly very short. This brief period of time suggests that future pandemics with similar characteristics to those of the COVID-19 pandemic permit only a narrow window for preemptive intervention.

## Supplementary Materials

science.sciencemag.org/content/372/6540/412/suppl/DC1 Materials and Methods Supplementary Text Figs. S1 to S18 Tables S1 to S7 References (*45*,*54*) MDAR Reproducibility Checklist Data S1
